# Community analysis of the abundance and diversity of mosquito species (Diptera: Culicidae) in three European countries at different latitudes

**DOI:** 10.1186/s13071-017-2481-1

**Published:** 2017-10-23

**Authors:** Tim W. R. Möhlmann, Uno Wennergren, Malin Tälle, Guido Favia, Claudia Damiani, Luca Bracchetti, Constantianus J. M. Koenraadt

**Affiliations:** 1 Laboratory of Entomology, Wageningen University and Research Centre, P.O. Box 16, 6700 AA Wageningen, The Netherlands; 2 IFM Theory and Modelling, Linköping University, 581 83 Linköping, Sweden; 3 Scuola di Bioscienze e Medicina Veterinaria, Università degli Studi di Camerino, 62032 Camerino, Italy

**Keywords:** Disease vectors, Community composition, Non-metric multidimensional scaling, Host-seeking behaviour, Vector surveillance

## Abstract

**Background:**

Studies on mosquito species diversity in Europe often focus on a specific habitat, region or country. Moreover, different trap types are used for these sampling studies, making it difficult to compare and validate results across Europe. To facilitate comparisons of trapping sites and community analysis, the present study used two trap types for monitoring mosquito species diversity in three habitat types for three different countries in Europe.

**Methods:**

Mosquitoes were trapped using Biogents Sentinel (BGS), and Mosquito Magnet Liberty Plus (MMLP) traps at a total of 27 locations in Sweden, the Netherlands and Italy, comprising farm, peri-urban and wetland habitats. From July 2014 to June 2015 all locations were sampled monthly, except for the winter months. Indices of species richness, evenness and diversity were calculated, and community analyses were carried out with non-metric multidimensional scaling (NMDS) techniques.

**Results:**

A total of 11,745 female mosquitoes were trapped during 887 collections. More than 90% of the mosquitoes belonged to the genera *Culex* and *Aedes*, with *Culex pipiens* being the most abundant species. The highest mosquito diversity was found in Sweden. Within Sweden, species diversity was highest in wetland habitats, whereas in the Netherlands and Italy this was highest at farms. The NMDS analyses showed clear differences in mosquito communities among countries, but not among habitat types. The MMLP trapped a higher diversity of mosquito species than the BGS traps. Also, MMLP traps trapped higher numbers of mosquitoes, except for the genera *Culex* and *Culiseta* in Italy.

**Conclusions:**

A core mosquito community could be identified for the three countries, with *Culex pipiens* as the most abundant species. Differences in mosquito species communities were more defined by the three countries included in the study than by the three habitat types. Differences in mosquito community composition across countries may have implications for disease emergence and further spread throughout Europe. Future research should, therefore, focus on how field data of vector communities can be incorporated into models, to better assess the risk of mosquito-borne disease outbreaks.

**Electronic supplementary material:**

The online version of this article (10.1186/s13071-017-2481-1) contains supplementary material, which is available to authorized users.

## Background

Intensified movement of humans, animals, and goods on a global scale in combination with climate change creates opportunities for invasive and often exotic Culicidae vector species to establish in Europe [1]. Even without the arrival of exotic mosquitoes, suitable vector species are already present and may facilitate the successful spread of pathogens [2–7]. The introduction of West Nile virus (WNV) in the USA is probably the most striking example of a pathogen that was rapidly spread by the local vector community throughout the entire country [8]. Moreover, outbreaks of WNV caused by mosquito vectors in Romania (1996) and Russia (1999) resulted in hundreds of human cases, although rapid spread throughout Europe was not observed [9].

Mild winters in combination with humid and hot summers allow vector populations to proliferate rapidly, resulting in increased mosquito nuisance and vectorial capacity [10]. Human cases of WNV in Europe were reported during 2016 for Spain, Italy, Austria, Romania, Hungary, Serbia and Ukraine [11]. The continued emergence of arboviruses in southern, eastern, and central Europe justifies the demand for detailed knowledge about the vectors that could transmit pathogens [3, 9, 12]. For example, a theoretical modelling study by Roche et al. [13] suggested that higher vector species richness can increase pathogen transmission. In contrast, a study by Chaves et al. [14] suggested that higher diversity in vector communities decreases the risk of amplification and spread of disease. To better understand the role of vector communities in disease spread, knowledge about vector species distribution, abundance, and richness is therefore essential.

In Europe, several mosquito species, including the *Culex pipiens* complex, *Cx. modestus* (Ficalbi, 1889), the *Anopheles maculipennis* complex, *Aedes vexans* (Meigen, 1830) and *Ae. albopictus* (Skuse, 1895), can act as vectors of parasites or viruses like malaria, Zika virus, West Nile virus, or Rift Valley fever virus [2, 4, 7, 15]. Thus far, ecological studies on vector species diversity often focused on one specific country [16–18], region within a country [4, 19], or even on a single habitat [14, 20–23]. In addition, mosquito species diversity has mostly been studied with one, rather than with a selection of different surveillance trap types. The use of different trap types in each study makes it difficult to make direct comparisons between them. Given the lack of standardized, cross-European studies, this study aimed to sample and assess mosquito species diversity simultaneously. This was done by using two mosquito trap types, in three representative countries at different latitudes across Europe, and for three different habitat types. With this setup, the differences in species richness, diversity, and community composition in different habitats across different countries in Europe could be identified. In addition, the relative efficiency of two trap types could be compared.

## Methods

### Mosquito sampling

Adult mosquitoes were sampled with two trap types: the Biogents Sentinel (BGS) trap (BioGents GmbH, Germany, http://www.biogents.com/) and the Mosquito Magnet Liberty Plus (MMLP) trap (Woodstream Corp., USA, http://www.mosquitomagnet.com/). For the production of CO_2_ in the BGS trap, a mixture of 17.5 g dry instant yeast (Bruggeman, the Netherlands), 250 g white granulated sugar and 2 l of tap water in a 5 l plastic bottle was used [24]. For the MMLP trap, combustion of propane provided CO_2_.

### Sampling locations

**Fig. 1:**
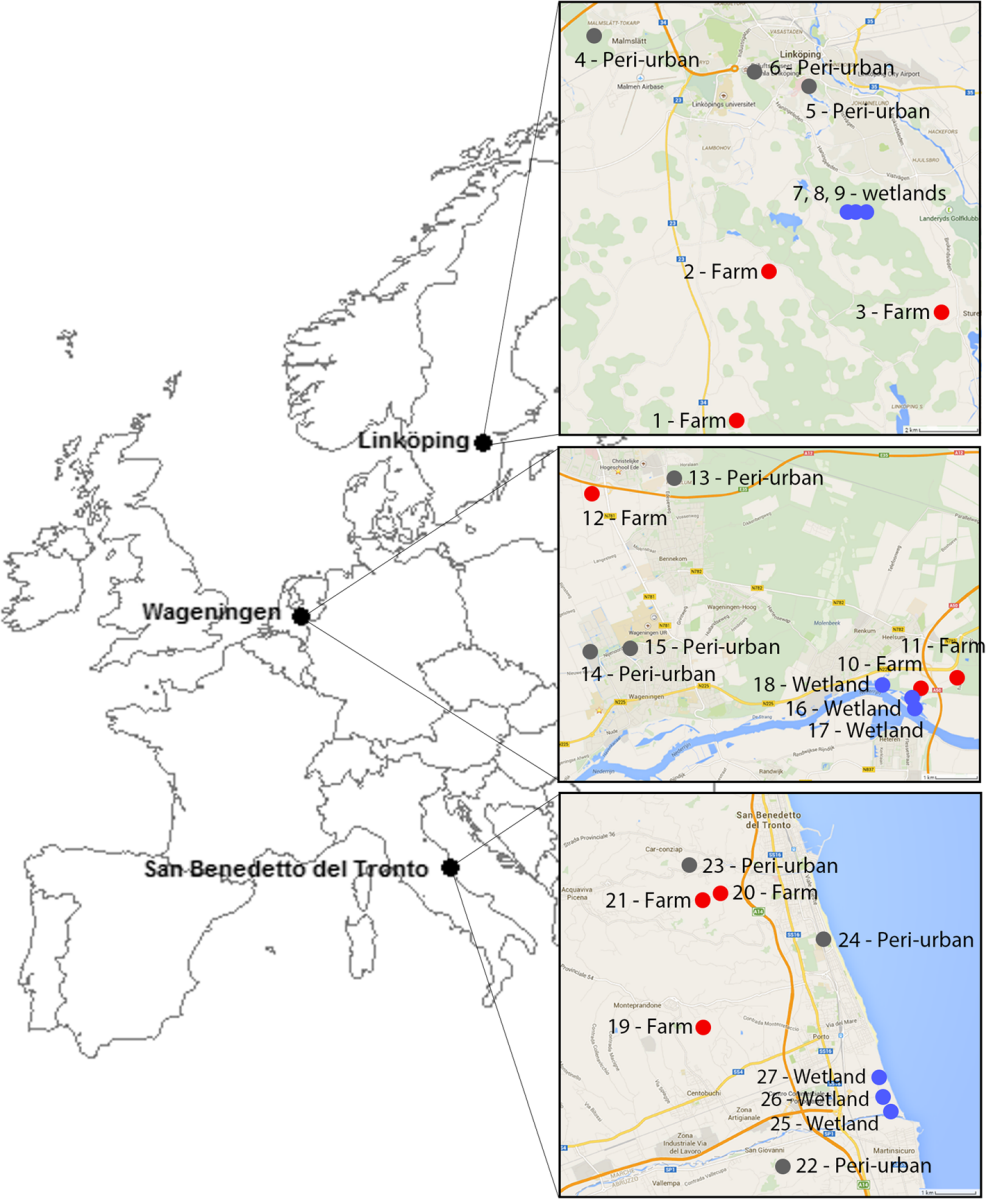
Overview of selected study sites. Overview of selected sites (1 to 27, see Vogels et al. [28] for more details about the locations) within each of the three countries in Europe: Linköping, Sweden (58.410808N, 15.621532E); Wageningen, the Netherlands (51.964795N, 5.662898E); San Benedetto del Tronto, Italy (42.949483N, 13.878503E). Farm habitats are indicated with a red dot (1–3, 10–12, 19–21), peri-urban habitats with a grey dot (4–6, 13–15, 22–24), and wetland habitats with a blue dot (7–9, 16–18, 25–27)

The traps were placed in three countries at different latitudes across Europe: southern Sweden (surroundings of Linköping 58.410808 N, 15.621532E, 45 m elevation), the central part of the Netherlands (surroundings of Wageningen 51.964795N, 5.662898E, 9 m elevation), and central Italy (surroundings of San Benedetto del Tronto 42.949483N, 13.878503E, 4 m elevation). In each country, three habitat types were sampled: (i) wetlands, (ii) farms, and (iii) peri-urban areas (Fig. 1). Wetlands are often considered as primary spots for transmission of vector-borne diseases as both reservoirs (birds), susceptible hosts (large grazers), and vectors (mosquitoes) can be present at a single location [23, 25, 26]. Farms were sampled because vector-borne diseases can have a large impact on livestock welfare, associated with high economic loss. Peri-urban areas are hypothesized to have a higher likelihood of human infection with a zoonosis, because of their location at the periphery of urban areas and proximity to farmland areas [27]. Each habitat type was represented by three unique sampling locations (Fig. 1), each separated by at least 100 m. At these locations, traps were placed at a minimum distance of 1 m from any walls or fences and were sheltered from the wind, rainfall, and direct sunlight as much as possible.

Trap locations in wetlands had a minimum of 50% marshy or standing water within a 100 m radius of the traps. The farms selected for sampling had at least 100 dairy cows, except for locations 10, 20 and 21 [28], which had a minimum of ten dairy cows. Traps were placed within 50 m of an open livestock stable present at the farm. Peri-urban locations were at the periphery of a city (inhabitants < 150,000). Within a 50 m radius of the trap, at least two occupied residential properties were present. Gardens were open, except for two locations (13 and 14 in the Netherlands) that were bordered on least at three sides of the garden with fences of 2 m height.

Habitat types matched the classification of the CORINE European Land cover database [29], although habitats classified as wetlands for the present study were on some occasions classified as ‘agricultural’ or ‘natural forest area’. One of the peri-urban sites in Italy was classified as ‘agricultural area’ instead of ‘artificial surfaces’ within the CORINE database, most likely because it was situated at the very edge of the city.

### Sampling procedures

Collections were performed monthly during six consecutive days in each country. Within each month, the exact timing of the sampling period varied for the three countries. Traps were active for 24 h and were emptied and rotated among the sampling locations (three trapping locations, in three different habitat types, and three countries) between sunrise and sunset of the next day. Sampling took place from July 2014 to June 2015, except for the winter months December, January and February (and March for Sweden). Mosquitoes were stored at -20 °C in Eppendorf tubes containing small silica beads covered with cotton wool.

### Sample identification

All female mosquitoes were identified to species level using the key of Becker et al. [30]. Morphologically similar species were recorded as belonging to a complex of one of the following species: *An. claviger*, *An. maculipennis*, *Ae. cantans*, *Ae. caspius*, *Ae. cinereus*, *Ae. detritus* and *Cx. pipiens*. These names are used throughout the remainder of the manuscript as a representative for all species in each complex. For the taxonomy of *Aedini* species, the classification of Becker et al. [30] and Wilkerson et al. [31] was used.

### Statistical analyses

Species diversity and evenness were calculated for the three countries and the farm, peri-urban, and wetland habitats. In addition, diversity indices were calculated for the two trap types (BGS and MMLP). Simpson’s Index of Diversity was calculated; $1-D=1-\frac{\sum {n}_i\left({n}_i-1\right)}{N\left(N-1\right)}$, where *n*_*i*_ is the number of the *i* ^th^ species and *N* is the total number of specimens in the studied country or habitat. Simpson’s Index of Diversity reflects the probability that two individuals taken at random from the dataset are not the same species. Values for Simpson’s Index of Diversity range between 0 and 1, with larger values representing greater diversity. The Shannon-Wiener’s Diversity Index was also used as a diversity index and calculated as ${H}^{\prime }=-\sum_{i=1}^R{p}_i\ln \left({p}_i\right)$, where ${p}_i=\frac{n_i}{N}$. The Shannon-Wiener’s Diversity Index is based on the uncertainty that an individual taken at random from the dataset is predicted correctly as a certain species. Larger values represent larger uncertainty, thus greater diversity. This method is sensitive to sample size, whereas the Simpson’s Index puts more weight on dominant species and is hardly influenced by a few rare species. In addition, the Shannon-Wiener’s evenness was calculated as $E=\frac{H^{\prime }}{\ln (S)}$, where *S* is the total number of species for the country or habitat. Values range between 0 and 1, where 1 is complete evenness, i.e. all species being equally abundant.

The effect of trap type on the number of mosquitoes per genera was analysed using a Mann-Whitney-Wilcoxon test, as these data were not normally distributed and variance was unequal. To better understand whether sufficient trapping efforts have been made for a reasonable estimate of species diversity, a rarefaction curve of the species and the number of collected mosquitoes were created with the rarecurve function within the VEGAN version 2.9.2. package [32] in R version 3.2.3 [33].

To examine the combined effect of country, habitat, and diversity on the mosquito community composition, non-metric multidimensional scaling (NMDS) analyses were performed. This method of data analysis creates a spatial ordination based on proximities between the elements of interest (habitat type, country, mosquito species, and mosquito abundance in this case) [34]. The degree of stress for each NMDS plot was calculated, which indicates the reliability of the outcome, i.e. lower stress corresponds with a higher reliability. The ordination of elements is considered arbitrary with stress values of 0.3 or above. The dissimilarity matrices are based on abundances for each species within the community. Distances between points were determined with the metaMDS function using the Bray-Curtis dissimilarity metric. All data were analysed in the statistical software package R version 3.2.3 [33].

## Results

A total of 887 trap collections were performed in Sweden, the Netherlands and Italy. In 617 (70%) of these collections, one or more mosquitoes were trapped. The BGS trap and MMLP trap ran effectively on 457 and 430 occasions, respectively.

A total of 11,745 mosquitoes were trapped during this study. Of these, 10,191 (87%) female mosquitoes could be identified to species level. Other individuals were either males (1376; 11.7%) or damaged (178; 1.5%) to the extent that they could not be identified morphologically. Over the three countries, a total of 40 mosquito species were found, comprising six genera. The rarefaction plots for each of the three countries are beyond their exponential growth curve, and level off (Additional file 1: Figure S1). This shows that our sampling effort was sufficient for obtaining a representative number of species for our locations in the three countries. The total number of female mosquitoes trapped during the field study in the three countries combined was highest for the genus *Culex* (61.6%), followed by *Aedes* (29.4%), *Culiseta* (4.7%)*, Anopheles* (3.2%), *Coquillettidia* (1.0%) and *Uranotaenia* (0.2%). The most abundant species was *Culex pipiens* with a total of 5202 (51%) out of all identified female mosquitoes (*n* = 10,191) from the three countries.

Overall, the MMLP trapped the largest numbers of mosquitoes in Sweden and Italy, while the BGS trapped most mosquitoes in the Netherlands. In all countries, the MMLP trapped most species and had the highest diversity in the collections trapped (Table 1). Of all 40 mosquito species trapped, 95% were found in the MMLP traps, whereas only 55% were found in the BGS traps.

**Table 1:** Species diversity indices by trap type. Values for Simpson’s Index of diversity, Shannon-Wiener’s diversity and Shannon-Wiener’s evenness for two trap types in three countries, Sweden, the Netherlands and Italy

Taxonomic diversity	Sweden		The Netherlands		Italy	
	BGS	MMLP	BGS	MMLP	BGS	MMLP
No. of samples (trapping nights)	138	136	159	153	160	141
No. of species trapped	14	29	8	12	16	24
No. of specimens trapped	270	1028	2397	899	2108	3489
Simpson’s Diversity Index	0.776	0.877	0.091	0.475	0.469	0.722
Shannon-Wiener’s diversity	1.874	2.377	0.225	0.942	1.033	1.611
Shannon-Wiener’s evenness	0.71	0.706	0.108	0.379	0.373	0.507

*Abbreviations*: *BGS* Biogents Sentinel trap, *MMLP* Mosquito Magnet Liberty Plus trap

As the study design was the same for all habitats and countries, we can compare mosquito abundances between the two trap types. From the comparisons between the two traps, the MMLP collected significantly more mosquitoes per 24 h in six out of twelve comparisons: *Aedes* mosquitoes in Sweden and Italy, *Anopheles* mosquitoes in Sweden, the Netherlands and Italy, and *Culiseta* mosquitoes in the Netherlands. The BGS trapped significantly more for two out of twelve comparisons: *Culex* and *Culiseta* mosquitoes in Italy (Additional file 2: Figure S2). In the remaining four comparisons, both traps collected equal numbers of mosquitoes.

Although the number of samples taken, and specimens trapped in Sweden was the lowest, the highest species diversity, richness, and evenness were found here compared to the other two countries. The lowest values for diversity were found in the Netherlands (Table 2). The species richness and diversity of the habitats differed among countries. In Sweden, most species were trapped in the peri-urban habitat, while most species were trapped at farms in the Netherlands, and wetlands in Italy. Farms had the lowest species richness both in Italy and Sweden, while peri-urban habitats had the lowest species richness in the Netherlands (Table 2). Species diversity was highest in Swedish wetlands, whereas it was highest at farms within the Netherlands. In Italy diversity was comparable among habitats (Table 2).

**Table 2:** Mosquito species diversity by country and habitat. Estimators of taxonomic diversity with values for Simpson’s Index of diversity, Shannon-Wiener’s diversity and Shannon-Wiener’s evenness for three habitats (farms, peri-urban and wetlands) in three countries (Sweden, the Netherlands and Italy)

Species	Sweden				The Netherlands				Italy				Total
	Farms	Peri-urban	Wetlands	Total	Farms	Peri-urban	Wetlands	Total	Farms	Peri-urban	Wetlands	Total	
No. of specimens trapped	258	213	827	1298	591	1541	1164	3296	265	501	4831	5597	10,191
No. of samples	91	91	92	274	99	105	108	312	98	101	102	301	887
No. of species trapped	13	24	19	29	11	5	10	14	13	14	21	26	40
Simpson’s Index of Diversity	0.739	0.753	0.849	0.885	0.524	0.035	0.153	0.217	0.646	0.611	0.706	0.737	0.699
Shannon-Wiener’s diversity	1.667	2.062	2.153	2.425	0.857	0.096	0.395	0.482	1.502	1.259	1.439	1.62	1.803
Shannon-Wiener’s evenness	0.65	0.649	0.731	0.72	0.358	0.06	0.171	0.183	0.586	0.477	0.473	0.497	0.489

From the 1298 mosquitoes trapped in Sweden, 29 species were identified. Of these mosquitoes, *Ae. pullatus* (Coquillett, 1904) (18%), *An. maculipennis* (16%), *Cx. pipiens* (16%), *Ae. detritus* (11%) and *Coquillettidia richiardii* (Ficalbi, 1889) (8%), were the most common species (Table 3). The 827 mosquitoes trapped from wetlands in Sweden were dominated by *Aedes* species, most notably *Ae. pullatus* (28%), *Ae. detritus* (16%) and *Ae. cinereus* (Meigen, 1818) (15%). For the 258 mosquitoes trapped on farms, the dominating species were *An. maculipennis* (44%), *Cx. pipiens* (19%), and *An. claviger* (14%), whereas the 213 mosquitoes trapped in peri-urban habitats were dominated by *Cx. pipiens* (46%), *Cq. richiardii* (11%), and *Culiseta annulata* (Schrank, 1776) (9%).

**Table 3:** Mosquito species abundance by country and habitat. List of mosquito species with number of specimens for each country (Sweden, the Netherlands and Italy) and habitat type (farms, peri-urban and wetlands)

Species	Sweden				The Netherlands				Italy				Total
	Farms	Peri-urban	Wetlands	Total	Farms	Peri-urban	Wetlands	Total	Farms	Peri-urban	Wetlands	Total	
*Aedes albopictus*	0	0	0	0	0	0	0	0	37	272	4	313	313
*Aedes behningi*	0	2	13	15	0	0	0	0	0	0	1	1	16
*Aedes berlandi*	0	0	0	0	0	0	0	0	0	1	0	1	1
*Aedes cantans*	0	4	3	7	0	0	0	0	0	0	0	0	7
*Aedes caspius*	0	1	0	1	0	0	0	0	5	9	1664	1678	1679
*Aedes cataphylla*	0	8	0	8	0	0	0	0	0	0	0	0	8
*Aedes cinereus*	0	3	120	123	0	0	50	50	0	0	4	4	177
*Aedes communis*	0	0	0	0	0	0	2	2	0	0	0	0	2
*Aedes detritus*	1	7	131	139	1	0	0	1	12	41	282	335	475
*Aedes geniculatus*	0	1	0	1	0	0	0	0	0	1	2	3	4
*Aedes hexodontus*	1	0	0	1	0	0	0	0	0	0	0	0	1
*Aedes impiger*	0	0	0	0	0	0	0	0	0	1	0	1	1
*Aedes intrudens*	1	1	0	2	0	0	0	0	0	0	4	4	6
*Aedes leucomelas*	0	0	2	2	0	0	0	0	0	0	0	0	2
*Aedes mercurator*	0	0	33	33	0	0	0	0	0	0	0	0	33
*Aedes pullatus*	0	4	228	232	0	0	0	0	0	0	0	0	232
*Aedes riparius*	0	0	2	2	0	0	0	0	0	0	0	0	2
*Aedes rossicus*	0	0	1	1	0	0	0	0	0	0	0	0	1
*Aedes vexans*	1	5	6	12	1	0	13	14	0	0	7	7	33
*Anopheles algeriensis*	0	1	0	1	0	0	0	0	0	0	0	0	1
*Anopheles claviger*	35	8	6	49	2	0	1	3	0	1	3	4	56
*Anopheles maculipennis*	114	2	96	212	3	1	6	10	7	2	3	12	234
*Anopheles plumbeus*	16	3	0	19	4	0	1	5	0	11	0	11	35
*Anopheles sacharovi*	0	0	0	0	0	0	0	0	1	0	1	2	2
*Coquillettidia richiardii*	4	23	76	103	1	0	2	3	0	0	1	1	107
*Culex laticintus*	0	0	0	0	0	0	0	0	4	0	27	31	31
*Culex martinii*	0	0	0	0	0	0	0	0	9	4	878	891	891
*Culex mimeticus*	0	1	6	7	0	0	0	0	0	0	0	0	7
*Culex modestus*	5	13	2	20	0	1	1	2	0	1	58	59	81
*Culex pipiens*	48	100	57	205	316	1514	1070	2900	149	148	1800	2097	5202
*Culex pusillus*	0	0	0	0	1	0	0	1	0	0	16	16	17
*Culex theileri*	0	2	0	2	0	0	0	0	2	0	41	43	45
*Culiseta alaskaensis*	1	1	0	2	0	0	0	0	0	0	0	0	2
*Culiseta annulata*	27	20	16	63	258	24	18	300	3	3	2	8	371
*Culiseta bergrothi*	4	1	0	5	3	0	0	3	0	0	0	0	8
*Culiseta longiarealata*	0	0	0	0	0	0	0	0	34	6	17	57	57
*Culiseta morsitans*	0	1	28	29	0	0	0	0	1	0	0	1	30
*Culiseta ochroptera*	0	1	1	2	0	0	0	0	0	0	0	0	2
*Culiseta subochrea*	0	0	0	0	1	1	0	2	1	0	0	1	3
*Uranotaenia unguiculata*	0	0	0	0	0	0	0	0	0	0	16	16	16

The Netherlands had the lowest species richness, with 14 species identified in the 3296 mosquitoes trapped during the study period. The most common species found were *Cx. pipiens* (88%) and *Cs. annulata* (9%) (Table 3). Both in the wetland (1164 mosquitoes) and peri-urban (1541 mosquitoes) habitats *Cx. pipiens* was the dominating species with 92% and 98% of the trapped mosquitoes, respectively. From the 591 mosquitoes trapped at farms *Cx. pipiens* (53%) and *Cs. annulata* (44%) were trapped in almost equal number.

A total of 26 species was identified from the 5597 mosquitoes trapped in Italy, of which *Culex pipiens* (37%), *Ae. caspius* (30%), and *Cx. martinii* (Medschid, 1930) (16%) were the most dominant (Table 3). Wetland habitats (4831 mosquitoes) were mostly populated by these three species. The 265 mosquitoes trapped at Italian farms were dominated by *Cx. pipiens* (56%), *Ae. albopictus* (14%) and *Cs. longiareolata* (Marcquart, 1838) (13%), whereas in peri-urban habitats *Ae. albopictus* was the most abundant species with 54% of the 501 mosquitoes trapped, followed by *Cx. pipiens* (30%) and *Ae. detritus* (8%).

**Fig. 2:**
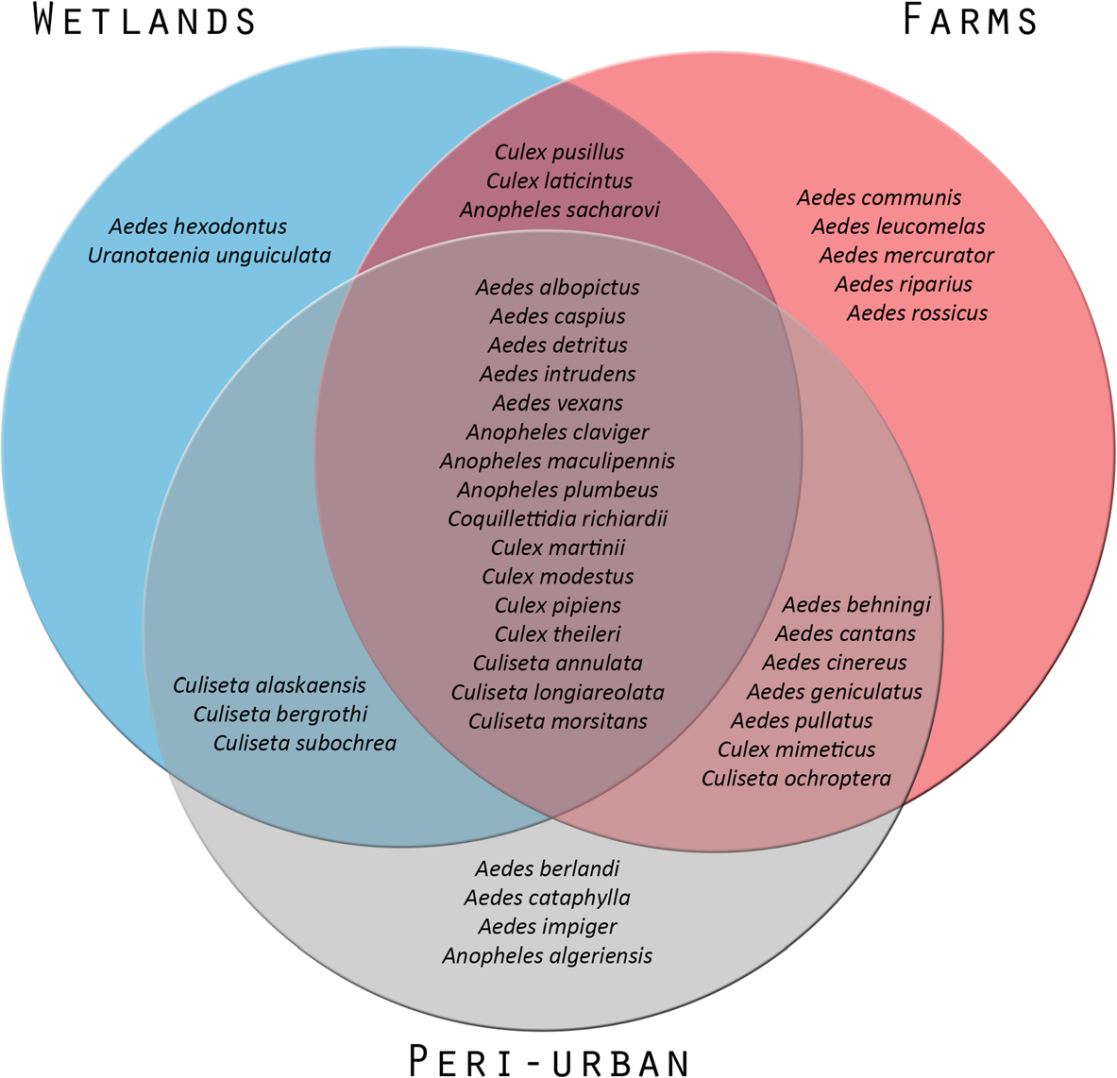
Venn diagram of habitats. Diagram shows the absolute presence of mosquito species found in farm (*red*), peri-urban (*grey*), and wetland (*blue*) habitats

**Fig. 3:**
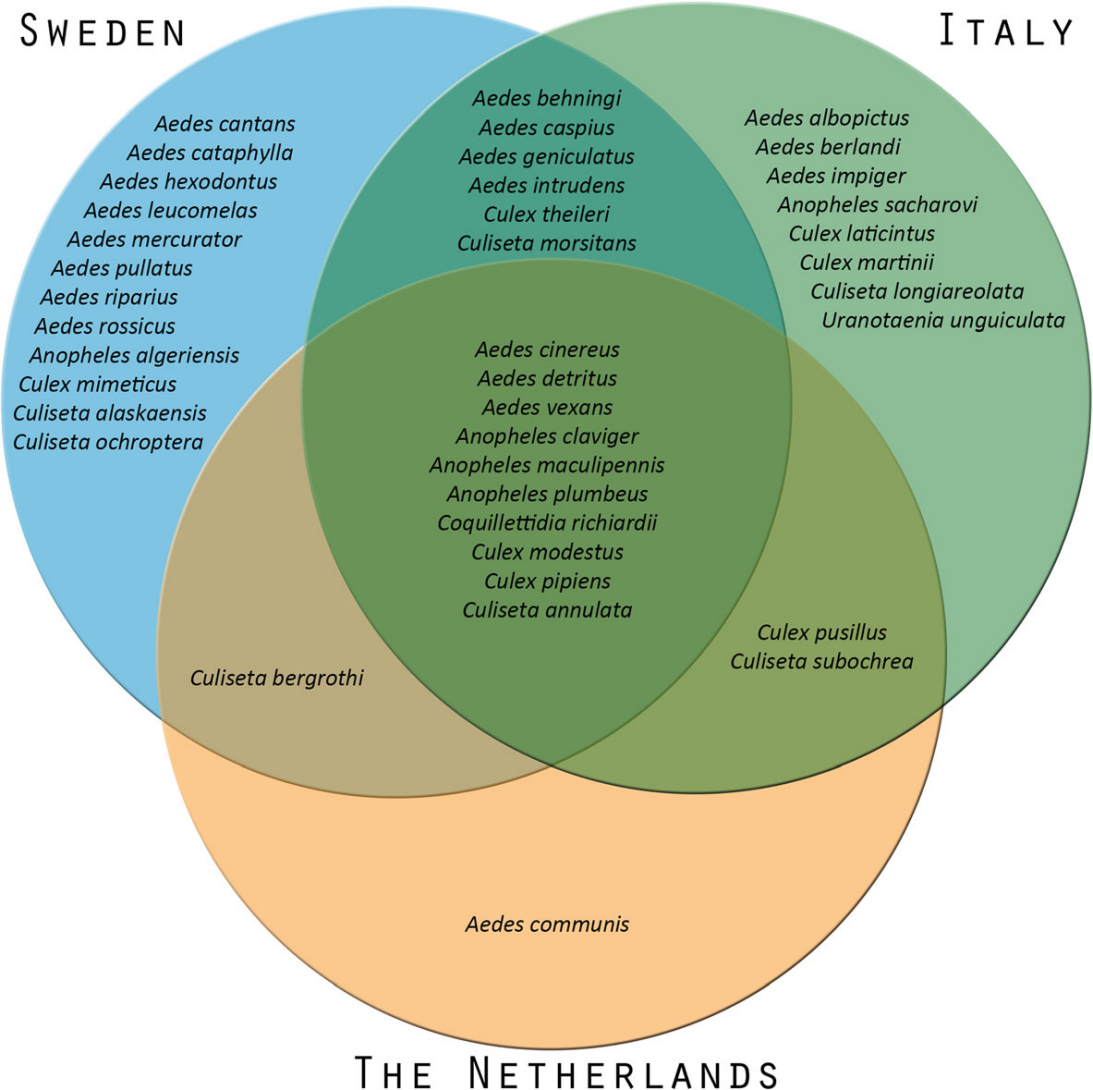
Venn diagram of countries. Diagram shows the absolute presence of mosquito species found in Sweden (*blue*), the Netherlands (*orange*), and Italy (*green*)

Most mosquito species (29/40, 73%) were found in at least two habitats. Five species occurred exclusively at farms, four species exclusively in peri-urban and two species exclusively in wetland habitats (Fig. 2). All these 11 species were found in one country only, indicating that they are unique trappings (Fig. 3). Furthermore, more than half of the 40 species were trapped in only one of the countries (21/40, 53%), while 25% (10/40) of the species were trapped in all countries (Fig. 3). The latter group included the most abundant species from the three countries: *An. maculipennis*, *Cx. pipiens*, *Ae. detritus*, *Cq. richiardii* and *Cs. annulata*. The most abundant mosquito in Sweden, *Ae. pullatus* only occurred in Swedish farm and wetland habitats. The second and third most abundant species from Italy (*Ae. caspius* and *Cx. martinii*) were trapped in all three habitats, but not in all countries (Figs. 2, 3).

**Fig. 4:**
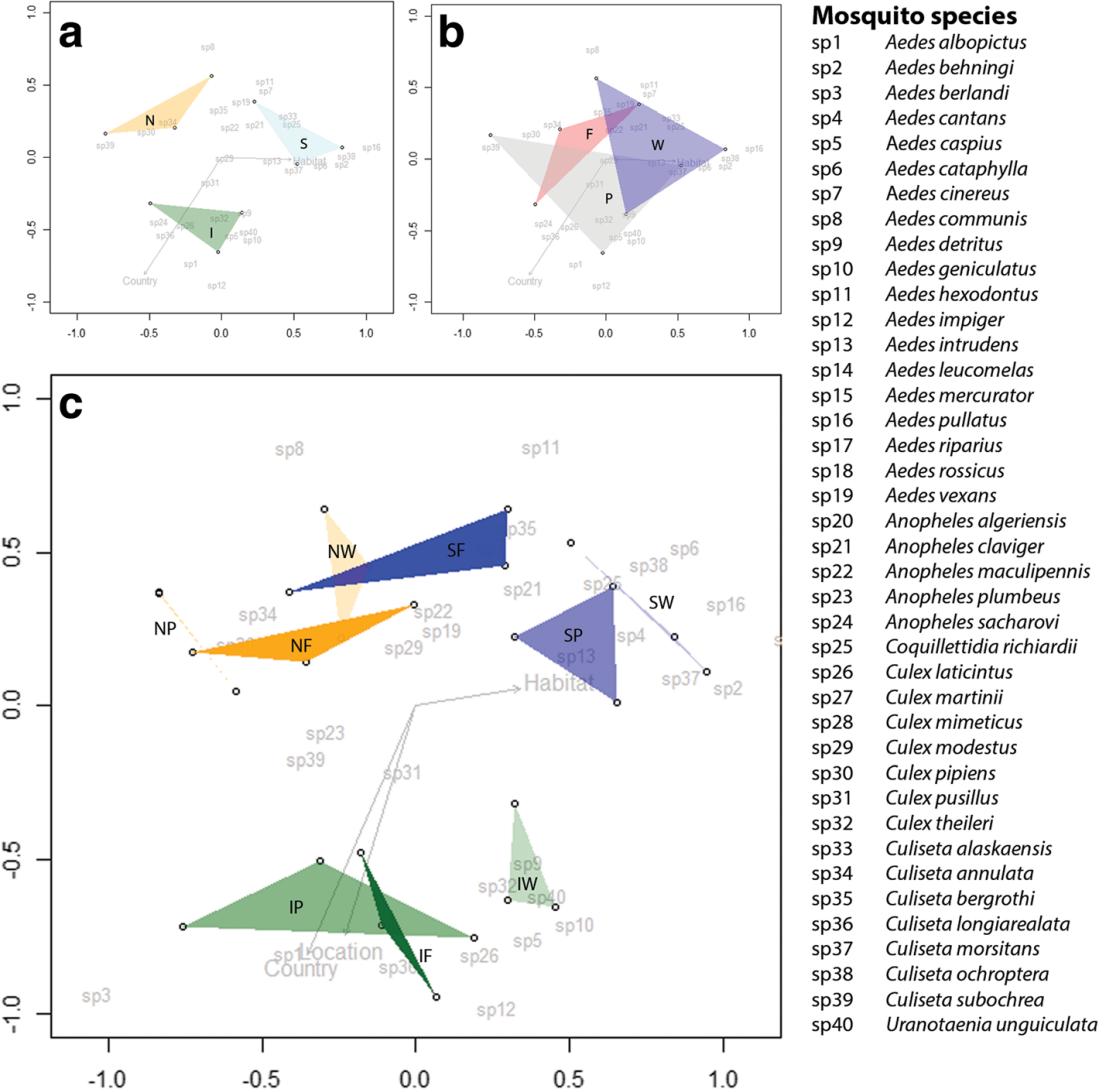
Results of NMDS analyses. **a** Mosquito community compositions for the three countries. *Abbreviations*: S, Sweden; N, the Netherlands; I, Italy. **b** NMDS analysis for the farm (F), peri-urban (P) and, wetland (W) habitats based on the number of mosquitoes trapped per species in each habitat and country. **c** NMDS analysis based on the number of mosquitoes trapped per species at each location in each country and habitat (Sweden in *blue*: SF, SP, and SW, the Netherlands in *orange*: NF, NP, and NW, Italy in *green*: IF, IP, and IW). The Bray-Curtis dissimilarity index was used for determination of dissimilarities among mosquito community compositions. Stress value = 0.119 for panels A and B, which indicates a good fit of the model. Stress value = 0.197 for panel C, which indicates a reasonable fit of the model

Dissimilarity matrices resulting from NMDS analyses reveal clear differences in mosquito community composition among countries (stress value = 0.119, *P* = 0.029) (Fig. 4a). No significant habitat differences among communities were found (stress value = 0.119, *P* = 0.537) (Fig. 4b). However, differences in mosquito communities among habitats within each country were found for some of the habitats (Fig. 4c). Habitat communities differed from each other in Sweden (stress value = 0.121, *P* = 0.03) and Italy (stress value = 0.088, *P* = 0.033), but were not significantly different from each other in the Netherlands (stress value = 0.041, *P* = 0.173).

## Discussion

To assess mosquito community diversity at a European scale, the present study used a standardized trapping protocol to sample mosquitoes in three countries at different latitudes across Europe. The highest mosquito diversity was found when trapping with the MMLP trap compared to the BGS trap. Although the BGS was initially developed for trapping host-seeking *Aedes* spp. [35], in the present study it did not trap large numbers of *Aedes* mosquitoes compared to the MMLP trap. On the other hand, it did trap significantly more *Culex* and *Culiseta* mosquitoes in Italy (Additional file 2: Figure S2). These results differ from the findings of a study in which four trap types in Germany were compared, and where the BGS was the most efficient trap [36]. However, that study used different attractive blends for each of their traps, possibly explaining the disparity with our results.

Although the CO_2_ used in the present study (via propane combustion or sugar fermentation) attracts mosquitoes [37], more specimens may be trapped if a lure or attractive blend is added to the traps [38, 39]. However, it is not clear whether there is a selective effect of specific blends on the attraction of different mosquito species. As CO_2_ is a general host-seeking cue for blood-feeding arthropods, only CO_2_ was chosen as an attractant for this study. Larval sampling could further complement adult female trapping to study mosquito diversity [17, 40, 41]. Furthermore, the number of trapped species and specimens can fluctuate substantially depending on the year [19]. Our data were collected for one year only, and do therefore not take into account annual variation in mosquito population dynamics.

Mosquito community composition differed among countries. This is illustrated by the diversity indices calculated (Table 2), which was highest in Sweden, followed by Italy and the Netherlands. Also, the Venn-diagram (Fig. 3) shows that 25% of the trapped mosquito species were found in all three countries. Finally, the dissimilarity matrix (Fig. 4a) distinguishes different mosquito communities among countries. However, a core community seemed to be present in all countries (Fig. 3). This core community includes the five most abundant species from the three countries: *An. maculipennis*, *Cx. pipiens*, *Ae. detritus*, *Cq. richiardii* and *Cs. annulata*. Although this core community occurs throughout the sampled countries, it cannot be assumed that their contribution to disease spread is similar in all countries. Species or biotypes within the *An. maculipennis* or *Cx. pipiens* complexes can, for example, differ in their feeding behaviour or vector competence [30, 42, 43] and thus play different roles in pathogen transmission.

In total, 29 of 49 mosquito species officially recorded for Sweden [17], 14 of 35 species for the Netherlands [18], and 26 of 54 species for Italy [44] were trapped during this study. Although our sampling effort was comprehensive, as can be seen in the rarefaction plot (Additional file 1: Figure S1), it should be mentioned that mosquito diversity in our collections is not representative for the countries as a whole. Results can be compared among the three countries in this study because of the consistent study design. However, sampling was done in a small representative area that cannot be extrapolated to the country level. Complete mosquito diversity for a country is better estimated with studies sampling throughout a country with many traps for a longer period, as can be illustrated by the fact that diversity indices found by Ibanez-Justicia et al. [18] are higher for the Netherlands than those in the current study (Table 2). Combining both setups for multiple countries and multiple years would be the ideal study design, but this is not logistically feasible. Mosquito species that were not trapped in this study most likely occur in very low densities or use different habitats than sampled in this study, thereby making them less relevant from the perspective of disease spread.

While community composition differed among countries, they overlapped among habitat types (Fig. 4b). However, when differentiating habitats within countries, there was marked habitat effects on community composition (Fig. 4c). Communities among habitats differed within Sweden and Italy, while communities in the Netherlands were more similar to each other for all habitats. This might be explained by the relatively high level of habitat fragmentation in the Netherlands [18]. As a result of high habitat diversity in the landscape on a small spatial scale, species may be more easily collected from nearby habitats.

Although diversity indices did not show a clear pattern for habitats (Table 2), species diversity was always higher in (semi-) natural areas (farms and wetlands) when compared to peri-urban habitats in all countries. This corresponds with other studies that found higher diversity in wet, inundated or heterogenic natural areas with a high vegetation index [14, 16, 21, 23, 41, 44, 45]. This probably reflects the fact that natural areas offer more diversity in breeding habitats, resting places, and available hosts for mosquitoes.

Although the fewest specimens were trapped in Sweden, the highest diversity was recorded here. It is accepted that species diversity in general, and also for mosquitoes, declines towards the pole regions [20, 45, 46]. However, if natural areas do indeed accommodate more mosquito diversity, this could explain the higher species diversity in Sweden. Also, high species richness in Sweden could be caused by the relatively high number of *Aedes* species trapped, as the tribe Aedini consists of more mosquito species than any of the other tribes in the Palaearctic [40].

An earlier study in Italian wetlands found 22 species, of which 14 overlapped with what we found in our Italian wetland site. However, their samples from the same location (Sentina wetlands, 42.901956N, 13.905395E) only comprised six out of the 21 species trapped within the present study [47]. The greater number of species trapped during our study compared to collections by Toma et al. [47], may be the result of the use of different trap types, and the further development and succession within the Sentina wetland natural area that was restored in 2004, as natural wetlands harbour more mosquito species than constructed wetlands [21].

From the European core mosquito community, several can be identified as (potential) vectors of pathogens. Species from the genera *Culex* and *Aedes* are known to transmit pathogens [30]. *Culex pipiens* was trapped in large numbers in all three countries and most of the habitats. Other studies in Europe also found *Cx. pipiens* to be one of the most dominant species [16, 23, 41, 44, 48, 49]. *Culex pipiens* is a known vector for WNV, which already circulates in some parts of Europe [2, 50]. It is still unclear why WNV does not spread to more northern countries in Europe [3, 9, 12], but the temperature seems to be one of the main driving factors [43, 51].

*Aedes albopictus* is a known vector of approximately 22 arboviruses, including WNV, dengue, chikungunya, and possibly Zika [15, 52–54]. In our study, they were mainly trapped from peri-urban sites in Italy where they even outnumbered *Cx. pipiens* (Table 3), but they were not found in Sweden or the Netherlands. However, *Ae. albopictus* is known to be repeatedly introduced into the Netherlands with the import of tires and lucky bamboo plants [55, 56]. It is expected that *Ae. albopictus* is unable to survive in Sweden, but that it can establish in the Netherlands [57]. The introduction and establishment of an efficient vector such as *Ae. albopictus* will significantly increase the risk of pathogen transmission, as was shown in Italy for outbreaks of chikungunya [58]. This stresses the need for appropriate monitoring and control strategies against this species.

Other *Aedes* species, such as *Ae. caspius*, *Ae. pullatus*, *Ae. detritus* and *Ae. cinereus*, were mainly trapped in Italian and Swedish wetlands. *Aedes caspius* is considered a potential vector for WNV and tularemia [30]. The high numbers of *Ae. caspius* mosquitoes trapped in Italian wetlands correspond to its association with brackish water in coastal wetlands [19, 23, 44, 49]. The *Ae. cinereus* mosquitoes, mainly trapped in Swedish wetlands, are considered an important bridge vector for both tularemia bacteria, and the Sindbis virus that is re-emerging in humans every seventh year in northern-European countries [17, 59]. Mosquitoes from the species responsible for maintaining the enzootic cycle of the Sindbis virus among birds, *Cx. torrentium* were only found in small numbers in our earlier study [28].

Besides the presence of specific vector species in the European mosquito core community, it is also important to take the diversity of communities associated with these dominant vectors into account [13, 14]. Mosquito community composition differed among countries and for some habitats within countries. Chaves et al. [14] suggest that higher diversity in vector communities is expected to decrease the risk of amplification and spread of a vector-borne disease because higher vector species diversity is thought to be correlated with lower mosquito abundance. In contrast, a theoretical study by Roche et al. [13] suggests that greater species richness can amplify disease transmission. Specific vector species could play an important role in these complex community dynamics. Given the fact that many vector-borne diseases require multiple species that together influence the rate of transmission, understanding the ecology of vector networks is becoming increasingly important.

## Conclusion

Within our study in three countries across Europe, a core mosquito community could be identified, with *Culex pipiens* as the most abundant species. Differences in mosquito community composition were more defined by countries than habitats, although some habitats do accommodate distinct communities in specific countries. Differences in vector community composition across countries may have implications for disease emergence and further spread throughout Europe. Both the role of these complex communities as well as the role of specific vector species within these communities should be further determined. To better understand patterns of disease emergence and outbreaks, differences in vector communities should, therefore, be incorporated in mathematical and statistical models.

## Additional files

Additional file 1: Figure S1. Rarefaction plot of sampling effort. The plot shows the number of species expected to be found for the number of individuals sampled for Sweden (blue), Italy (green) and the Netherlands (orange). (PNG 6 kb)

Additional file 2: Figure S2. Frequency distribution for the number of trap nights that a specific number of female mosquitoes was trapped. Shown are the results for three countries (Sweden, the Netherlands and Italy), two trap types (MMLP, Mosquito Magnet Liberty Plus trap; BGS, Biogents Sentinel trap), and the four most common genera (*Aedes*, *Anopheles*, *Culex* and *Culiseta*). Comparisons between the two trap types were made for the four most common mosquito genera in each country, using the Mann-Whitney-Wilcoxon test. Significance is displayed for each comparison, with *P < 0.05 and ***P < 0.001. (PNG 10822 kb)
