# Supplementary figures and images for: Community analysis of the abundance and diversity of mosquito species (Diptera: Culicidae) in three European countries at different latitudes
Source: Parasit Vectors. 2017 Oct 23;10:510. doi: 10.1186/s13071-017-2481-1 (PMC5653988; doi:10.1186/s13071-017-2481-1)

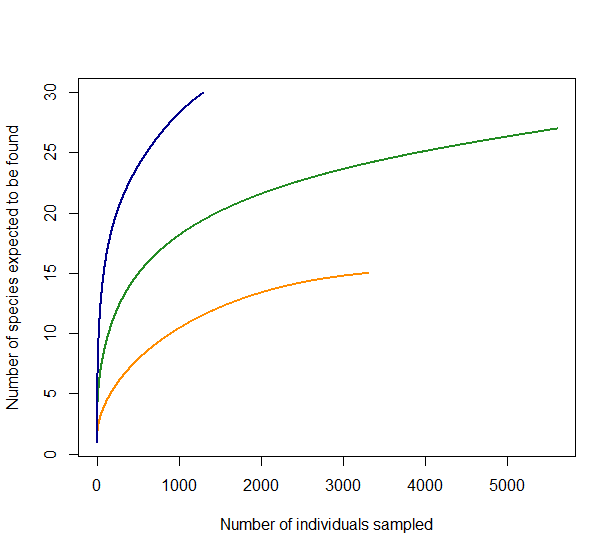

Supplement: Supplementary file 1 — Rarefaction plot of sampling effort. The plot shows the number of species expected to be found for the number of individuals sampled for Sweden (blue), Italy (green) and the Netherlands (orange). (PNG 6 kb) [file 13071_2017_2481_MOESM1_ESM.png]

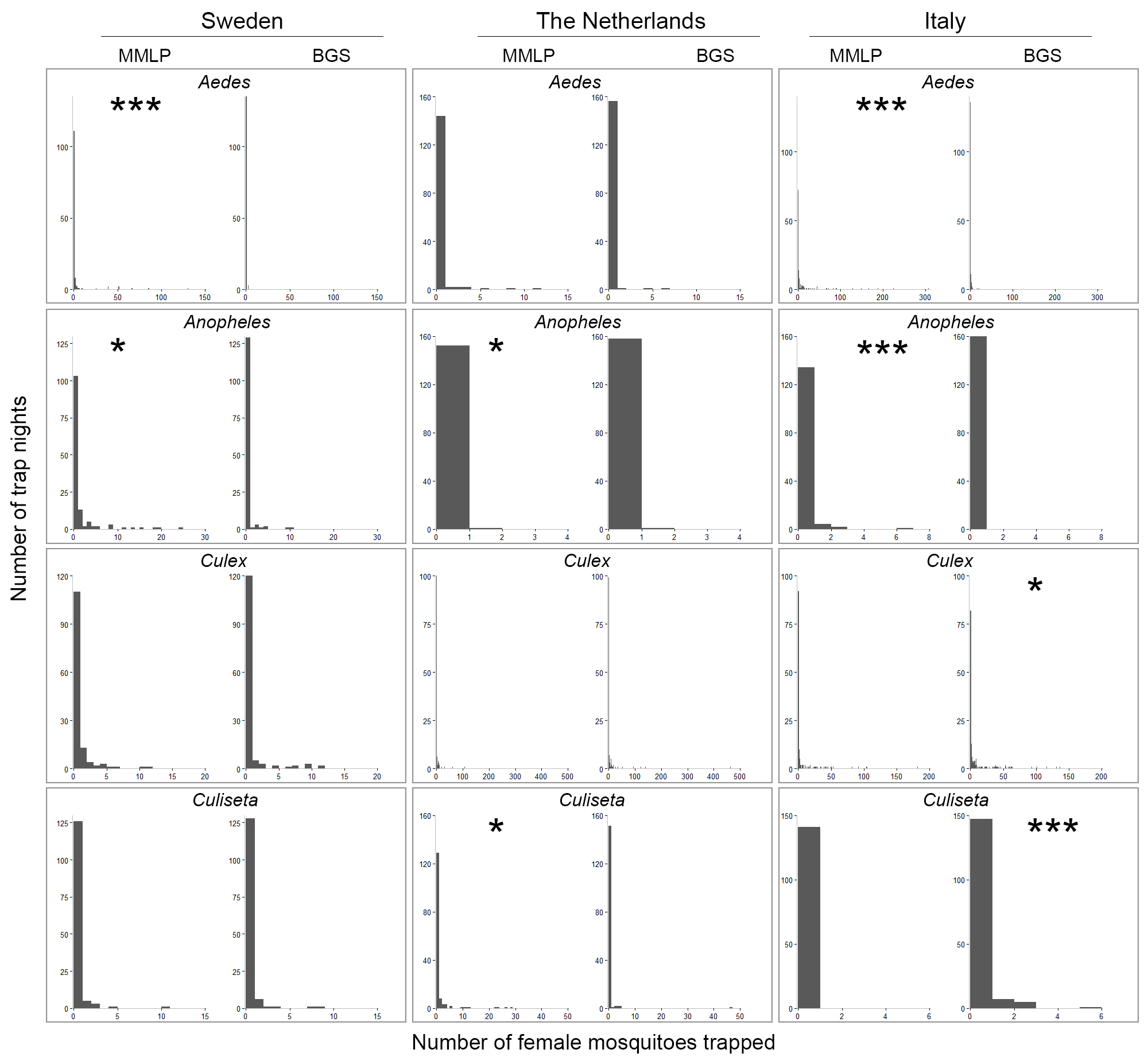

Supplement: Supplementary file 2 — Frequency distribution for the number of trap nights that a specific number of female mosquitoes was trapped. Shown are the results for three countries (Sweden, the Netherlands and Italy), two trap types (MMLP, Mosquito Magnet Liberty Plus trap; BGS, Biogents Sentinel trap), and the four most common genera (Aedes, Anopheles, Culex and Culiseta). Comparisons between the two trap types were made for the four most common mosquito genera in each country, using the Mann-Whitney-Wilcoxon test. Significance is displayed for each comparison, with *P < 0.05 and ***P < 0.001. (PNG 10822 kb) [file 13071_2017_2481_MOESM2_ESM.png]
